# New Method of Expelling the Tape Worm

**Published:** 1804-05-01

**Authors:** 


### New Method of expelling the Tape Worm.

Dr. DE BECK, Physician to the Emperor of Russia, has adopted the following method for expelling the Tape Worm, particularly that species with short and broad joints, (Taenia lata) The patient is to take, about four or five o'clock in the evening, the following powder, mixed with water or watergruel: ℞. Mercur. dulc. scr. j. corn. cervi usti, cinnabaris antimonii aa. gr. x. M. f. pulv. At night, after having taken some broth for supper, two ounces of the oil of sweet almonds are to be given. The next morning he is to take, before breakfast, *one* powder of the following mixture, which the Doctor calls *specificum*, in a tablespoon full of syrupus persicorum, after which he is to drink a cup or two of tea. ℞. Radic filicis maris dr. j. jalappæ, gummi guttæ, hb. cardui bened. corn. cerv. ust. aa. dr. ß. M. f. pulv. subtiliss. divid. in iij. partes æquales, s. specificum.

This powder generally occasions, within two or three hours, vomition, which however is much alleviated by drinking weak tea or some broth. The excrements discharged at the same time ought to be carefully examined, in order to see whether the whole tape worm, or only pieces of it have been voided; in which latter case the patient is to take a second powder; and should the whole tape worm not be expelled two or three hours after, the last dose of the specificum is to be given, by which the expulsion of the worm will certainly be effected. If the tape worm be voided early on the same day that the patient has taken the specificum, it generally shows signs of life on being put into a bason with warm water; but should it go off the day after, it is found dead in the stool.

To

To some patients the specificum neither causes vomition nor purging, though the worm is notwithstanding expelled. Sometimes the first dose of the specificum suffices for driving out the whole tape worm, but always succeeds with the second dose. In these cases the remedy occasions very little inconvenience to the patient, and he is generally able to walk about the following day and to attend to his usual occupations. But should the patient be obliged to take the last dose, the fasting and efforts of the stomach, the purging and the violent motions of the tape worm will produce debility and sickness of the stomach, which symptoms continue some time after, but are easily remedied. Young female patients, of a tender habit, have successfully gone through this cure, and often been well the following day, without having had recourse to any medical assistance.

Two hours after the expulsion of the tape worm, the patient is directed to eat something, and to take a glass of good wine; and also, if the worm should be voided, one hour after the last dose the patient may be allowed to take some food. The sickness of the stomach is soon removed by a cup or two of broth, or by a mixture of wine and water. As soon as the tape worm appears, care must be taken not to tear it off, for which purpose the piece hanging out may be wrapped in soft linen, or the patient ordered to sit on a flat vessel filled with four or five pints of luke warm milk, sweetened with sugar, into which agreeable liquor the worm will soon disentangle itself, particularly as the intestinal canal is impregnated with remedies highly disgusting to it. If the patient be weak and feels himself too much debilitated by the first dose of the specificum, the following doses may be diminished; and if the first does not sufficiently operate, one hour after he may be allowed to drink some broth. If the powder remains only one quarter of an hour in the stomach, its operation will certainly follow; but in case the remedy be immediately ejected, by the patient having an unsurmountable aversion to it, no operation is to be expected. Patients of a robust habit may take instead of the jalappa, hba. gratiolæ; and in case the tape worm does not go off the same morning, a clyster of bitter herbs saturated with Epsom salt may be administered. Should this however not sufficiently operate, the patient is to take the following powder in the space of three hours.—℞. Pulv. rad. jalapp. dr. j. hb. gratiolæ, scr. j. M. f. dos. jjj. D.

